# Effect of follicle size on pregnancy outcomes in patients undergoing first letrozole-intrauterine insemination

**DOI:** 10.1186/s40001-024-01794-8

**Published:** 2024-03-18

**Authors:** Li Ling, Di Xia, Yihan Jin, Renyun Hong, Jing Wang, Yuanjiao Liang

**Affiliations:** Reproductive Medicine Center, Zhongda Hospital, School of Medicine, Southeast University, Nanjing, 210009 People’s Republic of China

**Keywords:** Intrauterine insemination, Follicle size, Letrozole, Pregnancy rate, Live birth rate

## Abstract

**Background:**

Letrozole has been proven to be an effective method for inducing ovulation. However, little attention has been paid to whether the lead follicle size will affect the success rate of intrauterine insemination (IUI) with ovulation induction with alone letrozole. Therefore, we hope to investigate the effect of dominant follicle size on pregnancy outcomes on human chorionic gonadotropin (hCG) day of the first letrozole-IUI.

**Methods:**

A retrospective cohort study design was employed. We included patients with anovulation or unexplained infertility undergoing first IUI treatment with letrozole for ovarian stimulation. According to the dominant follicle size measured on the day of hCG trigger, patients were divided into six groups (≤ 18 mm, 18.1–19.0 mm, 19.1–20.0 mm, 20.1–21.0 mm, 21.1–22.0 mm, > 22 mm). Logistic models were used for estimating the odds ratios (ORs) with their 95% confidence interval (CIs) for achieving a clinical pregnancy or a live birth. A restricted cubic spline was drawn to explore the nonlinear relationship between follicle size and IUI outcomes.

**Results:**

A total of 763 patients underwent first letrozole-IUI cycles in our study. Fisher exact test showed significant differences among the six follicle-size groups in the rates of pregnancy, clinical pregnancy and live birth (P < 0.05 in each group). After adjusting the potential confounding factors, compared with the follicles ≤ 18 mm in diameter group, 19.1–20.0 mm, 20.1–21.0 mm groups were 2.3 or 2.56 times more likely to get live birth [adjusted OR = 2.34, 95%CI (1.25–4.39); adjusted OR = 2.56, 95% CI (1.30–5.06)]. A restricted cubic spline showed an inverted U-shaped relationship between the size of dominant follicles and pregnancy rate, clinical pregnancy rate, and live birth rate, and the optimal follicle size range on the day of hCG trigger was 19.1–21.0 mm. When the E_2_ level on the day of hCG trigger was low than 200 pg/mL, the clinical pregnancy rates of 19.1–20.0 mm, 20.1–21.0 mm groups were still the highest.

**Conclusions:**

The optimal dominant follicle size was between 19.1 and 21.0 mm in hCG-triggered letrozole-IUI cycles. Either too large or too small follicles may lead to a decrease in pregnancy rate. Using follicle size as a predicator of pregnancy outcomes is more meaningful when estrogen on the day of hCG trigger is less than 200 pg/ml.

**Supplementary Information:**

The online version contains supplementary material available at 10.1186/s40001-024-01794-8.

## Introduction

Intrauterine artificial insemination (IUI) after ovulation induction is often used for the treatment for infertile couples with anovulation, mild male factor infertility, endometriosis, or unexplained infertility [1, 2]. Many factors have been reported to affect the success of IUI treatment cycles [3–5]. One of these factors is the size of dominant follicle before triggering.

As we all know, the size of follicular is an indirect marker of oocyte maturity, the timing of human chorionic gonadotropin (hCG) trigger based on follicle size is crucial for the success of IUI cycles [6]. However, there is no general consensus on the ideal size of the follicle in the literature. A variety of studies have investigated the dominant follicle size in IUI cycles using ovulation induction drugs of clomiphene citrate (CC) or gonadotropins, yielding inconsistent findings. For instance, a study of 1483 CC-IUI cycles demonstrated that women with follicular diameters measured 20 mm or greater exhibited lower pregnancy rates compared to those with follicle diameters ranging from 15.00 to 19.99 mm [7]. Conversely, another study observed higher pregnancy rates when the leading follicles measured between 23 and 28 mm [8]. However, there is little clinical research foused on the effect of follicle size on pregnancy outcomes during letrozole-IUIs.

Letrozole, a frequently employed pharmacological agent in clinical settings for promoting ovulation, is classified as a third-generation nonsteroidal aromatase inhibitor. Its mechanism of action involves the reduction of estrogen levels in patients by impeding the conversion of androgens to estrogens, thereby stimulating the hypothalamus-pituitary gland to secrete increased levels of gonadotropins to facilitate ovulation [9]. In the treatment of unexplained infertility, letrozole has been shown to be as effective as clomiphene citrate with a lower risk of multiple births [10]. It is more effective to induce ovulation with letrozole than with clomiphene citrate in patients with polycystic ovary syndrome by using letrozole [11].

In the present study, we performed a retrospective clinical investigation to explore the effect of leading follicle size for hCG trigger on pregnancy outcomes in patients undergoing first letrozole-IUI.

## Materials and methods

### Study design and patients

A retrospective study was conducted at the Department of Reproductive Medicine Center, Zhongda Hospital, School of Medicine, Southeast University from January 2017 to October 2022.

This study included all women who underwent a first IUI cycle in our center after ovulation induction with letrozole. Each couple underwent fertility investigations that included semen analysis, ovulation assessment, as well as tubal patency testing. The inclusion criteria were as follows: at least one fallopian tube pantency, comfired by hysterosalpingography or laparoscopy; normal semen analysis parameters of the partner, according to WHO guidelines [12]. The study also included patients with one of the following infertility causes: ovulatory dysfunction, or unexplained factors. We excluded cycles that had incomplete medical records. Participants with multi-follicular development or a follicle stimulating hormone (FSH) value exceeding 12 IU/ml were not included in the study.

### Ethical approval

The study was approved by the Ethics Committee of Zhongda Hospital, Affiliated School of Medicine, Southeast University (2023ZDSYLL320-Y01). Informed patient consent was not required as the study was retrospective in nature and analyzed patient data anonymously.

### Ovarian stimulation protocols

Pelvic ultrasound and serum hormone levels, including FSH, luteinizing hormone (LH), and estradiol (E_2_), were assessed on the 2nd and 4th day of the menstrual cycle. Once the patients were confirmed to be in the early follicular phase, the administration of letrozole (Jiangsu Hengrui Medicine Co., China) at a dosage of 2.5–5 mg commenced on any of cycle days 3, 4, or 5 and continued for 5 days. A baseline ultrasound was conducted on the day of letrozole initiation, followed by subsequent ultrasounds 6–11 days later and then at intervals of 1–3 days as necessary until at least one follicle reached a mean diameter of ≥ 16 mm. In conjunction with ultrasound, the serum levels of E_2_, LH, and/or progesterone were assessed as necessary. Upon identification of a mature follicle, ovulation was induced using human chorionic gonadotropin (hCG; Lizhu Pharmaceutical Trading Co., China), and intrauterine insemination (IUI) was scheduled to take place within 24–36 h following hCG administration. In the event of an LH surge (defined as an LH level exceeding 2.5 times the baseline value) [13], IUI was scheduled for the subsequent day. Even though we didn't use hCG, we called it a "trigger day."

### Sperm preparation and insemination

Following a period of abstinence lasting 2–3 days, semen samples were collected via masturbation and subsequently subjected to a liquefaction process lasting 15–20 min. The density gradient centrifugation method was employed to perform sperm preparations. We finally calculated the post-preparation total motile count (TMC) of sperm [TMC = volume (mL) × count (10^6^ /mL) × percent motility]. During the IUI procedure, a single insemination was conducted per cycle. The patient assumed the bladder lithotomy position and proceeded to disinfect the vulva using normal saline. Subsequently, the vagina was washed, cervical mucus was removed, and the area was dried using aseptic dry gauze. A soft catheter (Cook Group, Bloomington, Indiana) containing 0.5 ml of sperm suspension was inserted into the fundus of the uterine cavity, retracted 1 cm, and then slowly injected until the entire suspension was administered. Following the procedure, patients remained in a supine position on the examination table for 20 min.

### Outcome measures

The primary outcome measured in this study was the live birth rate (LBR). The secondary outcomes were pregnancy rate (PR) and clinical pregnancy rate (CPR).

The pregnancy was characterized by a serum β-hCG concentration exceeding 5.73 mIU/ml, which was measured 14 days post-insemination. The clinical pregnancy was confirmed through ultrasonic visualization of at least one gestational sac within the uterus, exhibiting fetal cardiac activity, at the 6-week mark following IUI. Live birth (LB) was defined as the delivery of viable newborns occurring after the 28th week of gestation.

### Statistical analysis

Continuous variables were described as mean and standard deviation if they were normal distribution, otherwise, they were described as median and quartiles. Analysis of variance or Kruskal–Wallis test was used for comparisons between groups. Categorical variables were described as frequency and percentage, and Fisher’s exact approach was used to compare the difference among groups. Logistic models were used for estimating the odds ratios (ORs) and their 95% confidence interval (CIs) of pregnancy, clinical pregnancy rate and live birth respectively, with adjusting the potential confounding factors, including female age, body mass index (BMI), infertility type, the duration of infertility, infertility diagnosis, endometrium thickness, follicle-stimulating hormone (FSH), anti-Müllerian hormone (AMH), total motile count, luteinizing hormone (LH) surge and E2 on the day of hCG trigger. A restricted cubic spline was drawn to explore the nonlinear relationship between follicle size and clinical outcome. All statistical analyses were performed using R 4.1.3, and results with P-values less than 0.05 were considered statistically significant.

## Results

### Baseline characteristics

Between January 2017 and October 2022, a total of 763 patients underwent first letrozole-IUI cycles in our Center. The characteristics of these patients are described in Table 1. The mean age of the cohort was 30.70 years (SD = 3.80), the mean BMI ± SD was 22.86 ± 3.26 kg/m^2^. More than half (65.8%) of patients diagnosed ovulation dysfunction, and primary infertility occured in about 64.2% of patients. The median serum E2 level on the day of hCG trigger or LH surge in the entire cohort was 251.0 pg/mL, interquartile range 170.7–399.7 pg/mL. The mean ± SD endometrial thickness was 10.07 ± 2.15 mm. There were about 449 (58.84%) patients with endogenous LH surge who did not use hCG to trigger.

**Table 1:** General patient and cycle characteristics

Characteristic		
Age (y)		30.70 ± 3.80
BMI (kg/m^2^)		22.86 ± 3.26
Primary diagnosis	Anovulation	410 (53.7)
	Unexplained	353 (46.2)
Type of infertility	Primary infertility	490 (64.2)
	Secondary infertility	273 (35.8)
Duration of infertility (y)		2.39 ± 1.86
Cycle day 2–4 FSH level (mIU/mL)		7.88 ± 4.52
Serum AMH (ng/mL)		6.06 ± 4.72
Total motile sperm count TMC*10^6^		32.476 ± 14.28
Endometrial thickness (mm)		10.07 ± 2.15
E_2_ on the day of hCG trigger (pg/mL)		251.0 (170.7, 399.7)
LH on the day of hCG trigger (IU/L)		18.1 (10.7, 34.7)
LH surge	No	314 (41.15)
	Yes	449 (58.84)

### The association between pregnancy outcomes and the dominant follicle size

According to the diameter of the dominant follicle on the day of hCG trigger, patients were divided into six groups (≤ 18.0 mm, 18.1–19.0 mm, 19.1–20.0 mm, 20.1–21.0 mm, 21.1–22.0 mm, > 22.0 mm). The pregnancy outcomes of six groups are shown in Table 2, Fisher’s exact test showed significant differences among the six follicle-size groups in the rates of pregnancy, clinical pregnancy and LB (P < 0.05 in each case). The LBR of follicles 20.1–21.0 mm in diameter was the highest of 21.3%, follicles ≤ 18 mm in diameter group was the lowest of 9.57%.

**Table 2:** Pregnancy outcomes of different follicle size groups

Follicle size (mm)	N (%)	Pregnancy rate (%)	Clinical pregnancy rate (%)	Live birth rate (%)
Overall	763	18.84	17.69	14.42
≤ 18.0	188 (24.6)	12.23	12.23	9.57
18.1 ~ 19.0	114 (14.9)	14.92	14.03	11.40
19.1 ~ 20.0	161 (21.1)	25.47	23.60	19.88
20.1 ~ 21.0	108 (14.16)	23.15	23.15	21.30
21.1 ~ 22.0	78 (10.2)	24.36	23.08	16.67
> 22.0	114 (14.9)	14.04	13.16	9.65
P		0.008	0.012	0.007

We further performed stratified analysis by subgrouping patients according to E_2_ on the day of hCG trigger or LH surge (Additional file 1: Table S1 and S2). There were really no significant intergroup differences in pregnancy outcomes.

The odds ratios (ORs) and their 95% confidence interval (CIs) of pregnancy for different groups based on logistic models are shown in Table 3. After adjusting the potential confounding factors, including age, BMI, infertility type, the duration of infertility, infertility diagnosis, endometrium thickness, FSH, AMH, TMC, LH surge and E2 on the day of hCG trigger, compared with the follicles ≤ 18.0 mm in diameter group, 19.1–20.0 mm, 20.1–21.0 mm groups were 2.45 or 2.60 times more likely to get LB [adjusted OR = 2.45, 95%CI (1.30–4.61); adjusted OR = 2.60, 95%CI (1.31–5.14)]. Similarly, compared with the follicles ≤ 18.0 mm in diameter group, 19.1–20.0 mm and 20.1–21.0 mm groups were associated with an increase in the likelihood of pregnancy [adjusted OR = 2.57, 95%CI (1.45–4.56); adjusted OR = 2.20, 95%CI (1.17–4.16)], the clinical pregnancy [adjusted OR = 2.30, 95%CI (1.29–4.11); adjusted OR = 2.18, 95%CI (1.16–4.13)]. Although 21.1–22.0 mm group was associated with an increase in the likelihood of pregnancy and the clinical pregnancy, but not with LB. When compared with ≤ 18.0 mm group, > 22.0 mm group was not associated with the pregnancy outcomes.

**Table 3:** Odds ratios of pregnancy outcomes for different groups based on logistic models

Follicle size (mm)	N (%)	Pregnancy	Clinical pregnancy	Live birth
≤ 18.0	231 (30.3)	Ref.	Ref.	Ref.
18.1 ~ 19.0	71 (9.3)	1.33 [0.67, 2.65]	1.23 [0.61, 2.46]	1.27 [0.59, 2.73]
19.1 ~ 20.0	98 (12.8)	2.57 [1.45, 4.56]	2.30 [1.29, 4.11]	2.45 [1.30, 4.61]
20.1 ~ 21.0	171 (22.4)	2.20 [1.17, 4.16]	2.18 [1.16, 4.13]	2.60 [1.31, 5.14]
21.1 ~ 22.0	54 (7.1)	2.39 [1.20, 4.76]	2.20 [1.10, 4.42]	2.00 [0.91, 4.37]
> 22.0	138 (18.1)	1.23 [0.61, 2.47]	1.13 [0.56, 2.29]	1.09 [0.49, 2.43]

Additionally adjusted for Age, BMI, infertility type, duration of infertility, Primary diagnosis, endometrium thickness, FSH, AMH, TMC, E_2_ on the day of hCG trigger and LH surge

**Fig. 1:**
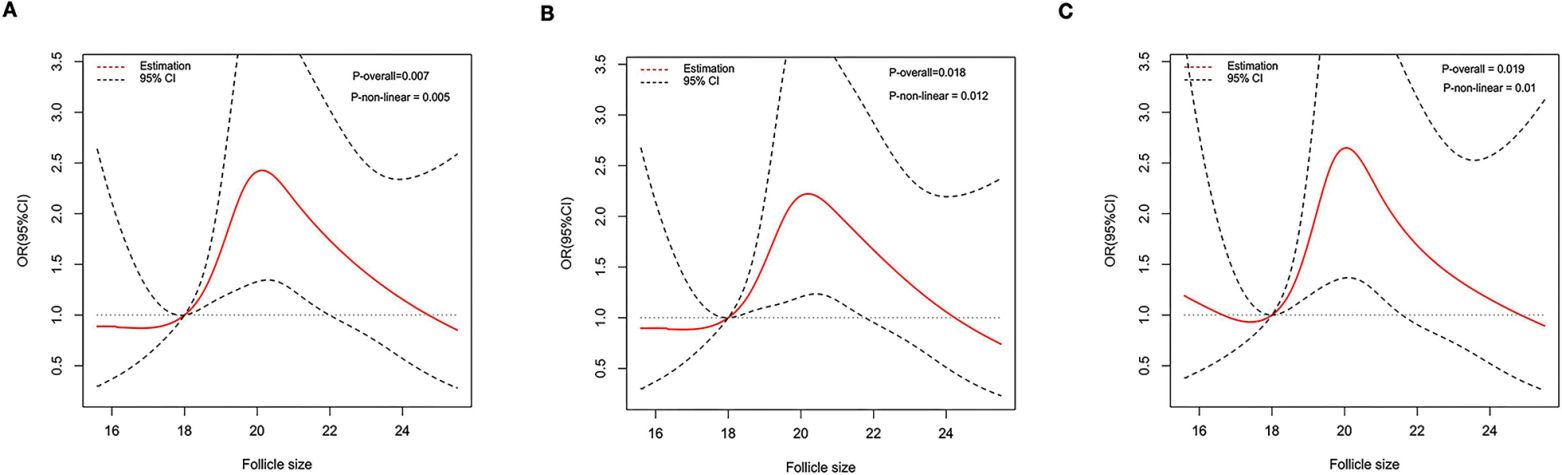
A restricted cubic spline showed an inverted U-shaped relationship between the size of primary follicles and pregnancy outcomes. **A** Pregnancy rate. **B** Clinical pregnancy rate. **C** Live birth rate. Additionally adjusted for Age, BMI, infertility type, duration of infertility, Primary diagnosis, endometrium thickness, FSH, AMH, TMC, E_2_ on the day of hCG trigger and LH surge

A restricted cubic spline showed an inverted U-shaped relationship between the size of primary follicles and pregnancy rate, clinical pregnancy rate, and live birth rate. The optimal follicle size range on the day of hCG trigger was 19.1–21.0 mm, and either too large or too small follicles may lead to a decrease in pregnancy rate (Fig. 1A–C).

### Subgroup analysis based on E2 level

On the basis of the different sizes of follicles group hierarchical analysis by serum E_2_ level on the day of hCG trigger. When the E_2_ level was low than 200 pg/mL, there was a significant difference among the six follicle-size groups in the rates of CR, and the 20.1–21.0 mm group had the highest CR of 22.5%. When the E_2_ level was higher than 200 pg/mL, there was no difference among the six follicle-size groups in the rates of CR (Table 4).

**Table 4:** Stratified analysis based on E_2_ levels on hCG trigger day, with clinical pregnancy as the outcome

Follicle size(mm)	E_2_ < 200 pg/ml 200 (n = 305)		E_2_ ≥ 200 pg/ml (n = 458)	
	Total	N (%)	Total	N (%)
≤ 18.0	69	5 (7.2)	119	18 (15.1)
18.1 ~ 19.0	59	7 (11.9)	55	9 (16.4)
19.1 ~ 20.0	71	16 (22.5)	90	22 (24.4)
20.1 ~ 21.0	45	10 (22.5)	63	15 (23.8)
21.1 ~ 22.0	27	5 (18.5)	51	13 (25.5)
> 22.0	34	2 (5.9)	80	13 (16.3)
χ2		11.33		5.61
P		0.045		0.346

### Sensitive analysis

We further performed four sets (< 18 mm, 18–20 mm, 20–22 mm and > 22 mm) of sensitivity analysis. When compared with < 18 mm group, the OR and 95%CI of LB for 18-20 mm and 20–22 mm groups were 1.93 [1.07, 3.49] and 2.33 [1.25, 4.32] (Additional file 1: Table S3), which were consistent with the main findings of our study.

## Discussion

In the present study, we found that in the first letrozole-IUI cycle, the optimal follicle size before hCG trigger was significantly associated with PR, CPR and LBR, even after adjusting for known confounders including female age, BMI, infertility type, the duration of infertility, infertility diagnosis, endometrium thickness, FSH, AMH, TMC, LH surge and E2 on the day of hCG trigger. The optimal follicle size range on the day of hCG trigger was 19.1–21.0 mm, which resulted in the highest PR, CPR and LBR. In addition, we also investigated the impact of estrogen levels on the day of hCG trigger on the outcome of the IUI cycle. When the E_2_ level was low than 200 pg/mL, there was a significant difference among the six follicle-size groups in clinical pregnancy rates. However, we did not observe a similar difference when the E_2_ level was more than 200 pg/mL.

Several studies have tested the predictive value of dominant follicle size on the success of IUI cycles. One study found that the most favorable lead follicle size in hCG-triggered letrozole-human menopausal gonadotropin IUI cycles ranged from 16.1 to 18.0 mm [14]. However, in another study, the size of the dominant follicle on hCG day in gonadotropin and IUI cycles did not demonstrate a statistically significant impact on pregnancy outcomes [15]. Inconsistent findings regarding the optimal size of lead follicles, ranging from 18 to 22 mm, have been reported in various studies [16–18]. Ghosh et al. discovered an inverse relationship between follicular diameter and pregnancy outcomes in clomiphene citrate IUI cycles. They observed that women with follicle sizes ranging from 15.00 to 19.99 mm had the highest likelihood of achieving pregnancy [7]. The authors proposed a classification system for follicles, categorizing them as immature (< 15 mm), mature (15–2 mm), and postmature (> 23 mm). Consequently, an excessive number of large follicles (≥ 20 mm) may decrease the chances of conceiving. Our study has drawn conclusions similar to this study, which also suggested either too large or too small follicles may lead to a decrease in pregnancy rate.

Although there is no general consensus on the ideal size of the follicle in the literature, the practice is that HCG is applied when the size is 18 mm or higher. Therefore, our study set < 18 mm as the smallest follicle group. Meanwhile, we also incorporated a greater number of follicle-size categories, aiming to more accurately evaluate pregnancy outcomes across a precise range of follicle sizes. Serial transvaginal ovarian ultrasounds with follicle measurement were performed by a fixed ultrasound physician in our study. The dominant follicle was measured from inner edge to inner edge to the nearest 0.1 mm in 2 perpendicular axes by transvaginal ultrasound. Our results showed the optimal follicle size ranging on the day of hCG trigger was 19.1–21.0 mm. In our study, although 21.1–22.0 mm group was associated with an increase in the likelihood of pregnancy and the clinical pregnancy, but not with live birth. Kolbe et al. also have found that the lead follicle size ranged from 21.1 to 22.0 mm was correlated with increased likelihood of clinical pregnancy among individuals undergoing their initial CC-IUI cycles, but unfortunately they did not follow-up the live birth rate [19]. Two other studies have also confirmed that follicles that are excessively small or excessively large will not produce mature oocytes and will not be suitable for fertilization [20, 21].

Ovulation induction drugs in previous studies were CC, or gonadotropins, CC combined with gonadotropins, or letrozole combined with gonadotropins, there is limited research on IUI for single letrazole. It was as effective as CC in inducing ovulation with letrozole. Rachmawati A et al. found that follicle sizes ranging from 18 to 22 mm in both the CC and Letrozole groups increased biochemical pregnancy rate. Furthermore, the use of Letrozole resulted in a 1.513 times higher biochemical pregnancy rate compared to CC [3]. These findings suggest that follicle sizes within the 18–22 mm range and the utilization of Letrozole as an ovarian stimulator are predictive factors for a higher pregnancy rate in women undergoing IUI.

Letrozole has gained widespread usage in the controlled ovarian hyperstimulation treatment of unexplained infertility and is now the preferred method of ovarian induction in anovulatory women with polycystic ovary syndrome (PCOS) [11, 22]. As a nonsteroidal aromatase inhibitor, letrozole exerts an antiestrogenic systemic effect by inhibiting the production of estradiol. In letrozole cycles, it is common to observe the presence of a mature follicle during the late follicular phase through ultrasound assessment, concurrent with a subphysiologic serum estradiol level, as granulosa cell aromatase activity has not yet rebounded from inhibition [23]. Therefore, we investigated the effect of E_2_ levels on the outcome of the IUI cycle on the day of hCG trigger. When we performed stratified analysis by subgrouping patients according to the levels of E_2_ on hCG-trigger day, there was no significant difference in pregnancy outcomes between the group with low and higher estradiol concentrations. Interestingly, when the E_2_ level was low than 200 pg/mL, there was a significant difference among the six follicle-size groups in pregnancy outcomes. However, when the E_2_ level was higher than 200 pg/mL, there was no difference. The potential anti-fertility effects of lower E_2_ in the ovulation induction treatment with letrozole, could include effects on the endometrium, periovulatory uterine contractions, fallopian tube function, and oocyte quality [24]. We just guess that high estrogen levels mean granulosa cell aromatase recovered from aromatase inhibition. When the follicle was with high estrogen concentration in letrozole cycle, the leading follicle size on the day of triggering would not affect the success of IUI. Therefore, we can speculate that using follicle size as a predicator of pregnancy outcomes is more meaningful when estrogen on the day of hCG trigger is less than 200 pg/ml. The potential impacts of letrozole with regard to its resulting lower E_2_ levels remain to be further studied.

A strength of this study is to only include patients diagnosed with unexplained infertility and anovulation, and to include the first IUI cycle with letrozole alone. This study distinguishes itself from previous research by incorporating a greater number of follicle-size categories, enabling a more precise assessment of pregnancy outcomes across a precise range of follicle sizes. The assessed endpoint of this study was live birth, which provided a more precise measure of the success rate of IUI. The current study is limited by its retrospective study design, which introduces the potential for confounding bias due to unmeasured confounding variables. In addition, the size of the follicles was determined by averaging the diameters of the follicles in 2 perpendicular axes in this study. For this reason, our data may not be applicable for the main follicle sizes measured using different techniques, and the variability of follicle size measurements might be a confounding factor in this study. And also it is a single center study with small sample size, so this population was underrepresented in the results.

## Conclusion

In conclusion, the optimal leading follicle size associated with better pregnancy outcomes ranges from 19.1 to 21.0 mm in individuals undergoing their initial letrozole-IUI cycles trigged by hCG, especially when estrogen on the day of hCG trigger is less than 200 pg/ml. According to present results, healthcare providers can determine the timing of HCG trigger based on the follicle size. Nevertheless, it is important to note that this study is retrospective in nature, thus necessitating additional well-designed prospective cohort studies to validate the findings.

### Supplementary Information

**Additional file 1: Table S1.** Stratified analysis based on LH surge on hCG trigger day. **Table S2.** Stratified analysis based on E_2_ levels on hCG trigger day. **Table S3.** Four sets of sensitivity analyses.

## Data Availability

The data that support the findings of this study are available on request from the corresponding author, Yuanjiao Liang, upon reasonable request.

## Abbreviations

IUI: Intrauterine insemination
hCG: Human chorionic gonadotropin
OR: Odds ratio
CI: Confidence interval
CC: Clomiphene citrate
FSH: Follicle stimulating hormone
LH: Luteinizing hormone
E_2_: Estradiol
TMC: Total motile count
PR: Pregnancy rate
CPR: Clinical pregnancy rate
LBR: Live birth rate
BMI: Body mass index
AMH: Anti-Müllerian hormone
