# Supplementary material for: Effect of follicle size on pregnancy outcomes in patients undergoing first letrozole-intrauterine insemination
Source: Eur J Med Res. 2024 Mar 18;29:184. doi: 10.1186/s40001-024-01794-8 (PMC10949705; doi:10.1186/s40001-024-01794-8)
Supplement: Supplementary file 1 — Additional file 1: Table S1. Stratified analysis based on LH surge on hCG trigger day. Table S2. Stratified analysis based on E2 levels on hCG trigger day. Table S3. Four sets of sensitivity analyses. [file 40001_2024_1794_MOESM1_ESM.docx]

**Table S1 Stratified analysis based on LH surge on hCG trigger day**

| LH surge | Pregnancy rate（%） | Clinical pregnancy rate（%） | Live birth rate（%） |
| --- | --- | --- | --- |
| No | 57 (18.15) | 55 (17.52) | 40 (12.74) |
| Yes | 84 (18.71) | 80 (17.82) | 70 (15.59) |
| χ2 | 0.038 | 0.011 | 1.218 |
| P | 0.846 | 0.914 | 0.270 |

**Table S2 Stratified analysis based on E_2_ levels on hCG trigger day**

| E_2_ levels on hCG trigger day | Pregnancy rate（%） | Clinical pregnancy rate（%） | Live birth rate（%） |
| --- | --- | --- | --- |
| <200 | 48 (15.74) | 45 (14.76) | 36 (11.80) |
| ≥200 | 93 (20.31) | 90 (19.65) | 74 (16.16) |
| χ2 | 2.536 | 3.014 | 2.813 |
| P | 0.111 | 0.083 | 0.094 |

**Table S3 Four sets of sensitivity analyses**

| Follicle size(mm) | Pregnancy | Clinical pregnancy | Live birth |
| --- | --- | --- | --- |
| ≤18 | Ref | Ref | Ref |
| 18.1~20.0 | 2.02 [1.18, 3.44] | 1.83 [1.07, 3.13] | 1.93 [1.07, 3.49] |
| 20.1~22.0 | 2.26 [1.29, 3.97] | 2.17 [1.23, 3.83] | 2.33 [1.25, 4.32] |
| >22.0 | 1.22 [0.61, 2.46] | 1.12 [0.55, 2.28] | 1.09 [0.49, 2.43] |

Additionally adjusted for Age, BMI, infertility type, duration of infertility, Primary diagnosis, endometrium thickness, FSH, AMH, TMC, E_2_ on the day of hCG trigger and LH surge
